# Rising up: Fertility trends in Egypt before and after the revolution

**DOI:** 10.1371/journal.pone.0190148

**Published:** 2018-01-18

**Authors:** Emma Radovich, Atef el-Shitany, Hania Sholkamy, Lenka Benova

**Affiliations:** 1 Faculty of Epidemiology and Population Health, London School of Hygiene & Tropical Medicine, London, United Kingdom; 2 Population & Family Planning Division, Ministry of Health & Population, Cairo, Egypt; 3 Social Research Center, The American University of Cairo, Cairo, Egypt; University Hospital of Münster, GERMANY

## Abstract

In 2014, Egypt’s Demographic and Health Survey (EDHS) documented an increase in the total fertility rate (TFR) to 3.5, up from a low of 3.0 recorded by the 2008 EDHS. The increase has been anecdotally attributed to the social upheaval following Egypt’s January 2011 revolution, but little is known about when fertility first began to increase and among which sub-groups of women. Using birth histories from seven rounds of EDHS (1992–2014), this study reconstructed fertility rates for single years from 1990–2013 and examined patterns of childbearing in five-year birth cohorts of women. We found that the decline in fertility reversed in 2007, earlier than postulated, plateaued and then increased again in 2013. The increase in TFR coincided with a convergence of fertility rates across education levels, and there is evidence of a shift toward childbearing at younger ages among more educated women, which may be inflating period measures of fertility.

## Introduction

Much has been written on the contribution of previously high fertility to the "youth bulge" in Egypt and its relationship to the 2011 revolution—part of a series of social upheavals across North Africa and the Middle East coined the "Arab Spring" [1,2]. Less is known about the recent increase in Egypt’s total fertility rate (TFR) [3]. Though the pace of decline may slow at later stages, the transition from high-fertility to low-fertility regimes is generally understood to be a one-way street: once started, fertility declines tend to continue [4,5]. The recent increase in fertility in Egypt is an unusual and worrying change of direction for the most populous country in the Arab world, whose population has doubled since the early 1980s to reach 84 million in 2014 [3]. This study presents the first detailed analysis of fertility trends before and after the revolution using multiple rounds of Egypt Demographic and Health Surveys (EDHS) to identify the pattern of change and sub-groups driving the increase in fertility rates.

### Background

In 1965, Egypt became the first country in the Arab world to launch an official family planning program [6,7]. Despite the program’s early establishment, political support was inconsistent and fertility remained high until the 1980s. Former President Hosni Mubarak was a strong supporter of family planning to lower fertility, seeing rapid population growth as hindering socio-economic progress [6]. Family planning under his regime was supported by large international investments, particularly from the U.S. Agency for International Development (USAID), which became the program’s largest foreign donor [7]. Since the first World Fertility Survey (predecessor to the EDHS) in 1980, Egypt’s TFR declined from 5.3 to 3.0 in 2008. The speed of fertility decline since the 1980s noticeably plateaued in the early 2000s and drew concern from population officials as to when—or whether—Egypt would reach replacement fertility [8–10].

In early 2011, mass protests in Cairo, Alexandria and other cities led to the ousting of Mubarak, who had ruled Egypt for 30 years, and widespread political instability. In the wake of three tumultuous years, results from the 2014 EDHS showed a considerable increase in fertility levels. The TFR recorded in the three years preceding 2014 increased to 3.5 from a historic low recorded in the 2008 EDHS of 3.0—reversing a 25-year trend of fertility decline [3,7]. The recent reversal of the trend has prompted alarmist pieces in popular media as to what the fertility increase spells for Egypt’s economic, environmental and political future [11–13].

The rise in fertility rates is widely believed to be linked to post-revolution social and political upheaval, potentially due to disruptions in family planning services or an increasing proportion of young women married in response to safety concerns—a factor that has been observed during periods of conflict in other Middle Eastern countries [14]. Postponement and declines in the proportion of women married have played significant roles in the transition to lower fertility across much of the Arab world as early marriage lengthens exposure to the risk of pregnancy and is associated with higher levels of childbearing [15,16]. Marriage remains nearly universal and divorce relatively rare in Egypt, and virtually all childbearing occurs within marriage [15,17]. The mean age at first marriage among women has increased in Egypt, following declines in adolescent marriage, but there is some evidence to suggest that this trend in marriage postponement has stalled or reversed among recent birth cohorts and may be contributing to the increase in fertility rates [17].

As a cross-sectional measure, TFR reflects the interaction between both timing (tempo) and level (quantum) of childbearing, and its sensitivity to changes in tempo of childbearing in the absence of a change in quantum is well documented [18]. During periods where women shift childbearing toward younger ages, the TFR is elevated, despite no actual change in completed family size. However, if the increase in TFR instead represents a change in quantum, this presents a worrying change of direction for Egyptian fertility and, if sustained, could have dramatic implications for future population growth. With more than 80 million people, Egypt’s rapid annual population growth rate of 2.6% threatens economic development, the environment (particularly regarding serious shortages of fresh water) and provision of services, including education and healthcare [19]. By 2030, Egypt's population is projected to reach 120 million [20]. Understanding the nature of the recent increase in TFR is thus critical to informing population policy.

This study reconstructs fertility trends in Egypt for the last quarter century to document when TFR first began to increase. Within the limits of the data, the study explores whether the increase in Egypt’s TFR represents a likely tempo change—in particular a concentration of childbearing at younger ages—rather than a quantum increase. Trends in the timing of childbearing by women’s birth cohorts through analysis of age at first marriage and mean number of children ever born at women’s exact ages is examined. Further, this analysis explores fertility patterns among certain sub-groups of women, in particular the urban and more educated, to investigate the impact of recent socio-political upheaval and changes in women’s educational opportunities on childbearing decisions.

The link between increased education and lower fertility is well established, and Egypt has made great strides in women’s education over the last 10 years. However, Bongaarts (2003) demonstrated that fertility differentials by education level tend to diminish at later stages in the fertility transition. The stalling fertility decline in Egypt observed in the early 2000s has been suggested to be related to the significant proportions of women with little or no education at the time [5]. While education is still far from universal, the 2014 EDHS documented substantial advancements: 52% of ever-married women age 15–49 had completed secondary school compared to 31% in 2000 and 20% in 1992 [3,21,22]. More educated women tend to marry at older ages than those with little or no formal schooling, though the differences across education levels is less pronounced in Egypt than in other Arab countries [16,17,23]. This study examines differentials in fertility rates by education level and how they have changed over time, with a particular focus on women with secondary school or higher education due to its effect on marriage age and subsequent fertility, regardless of financial position [10,24].

## Materials and methods

This study used retrospective birth history data from seven rounds of the EDHS: 1992, 1995, 2000, 2003 (interim survey), 2005, 2008 and 2014 to explore childbearing trends from 1990 to 2013. The EDHS collects nationally representative data on fertility and health indicators from ever-married women age 15–49. The seven datasets were appended to a single file of 107,138 ever-married women to create the analysis sample, enabling calculation of fertility rates by single calendar years and age-specific fertility rates (ASFRs) within sub-groups and by women’s five-year birth cohorts.

### Population coverage

The EDHS is designed to provide nationally representative estimates of population and health indicators for the whole country and the six regional subdivisions (Urban Governorates, urban and rural Lower Egypt, urban and rural Upper Egypt and the Frontier Governorates). The 1992 and the 2003 interim EDHS did not include the Frontier Governorates, which represent less than 2% of the population [22,25]. Due to security issues, the 2014 EDHS did not include North and South Sinai, two of the five governorates in the Frontier Governorates [3]. Each of the seven surveys used a multi-stage sampling design, and all ever-married women age 15–49 who were regular residents or present in the sampled households the night before the interview were eligible for inclusion. For each survey year, interviews were conducted over a period of 2–3 months and all surveys had a response rate greater than 98%.

### Sampling design

Women’s individual observation weights provided in DHS datasets to account for under- or over-sampling of sub-groups are relative to each other within each survey round. In order to use observations collected in various surveys, these weights were adjusted to reflect survey-specific weights and also to take into account the relative size of the sample in each survey year to the total population of interest [26]. A de-normalized weight variable was created for each survey separately by multiplying the individual weight variable by a coefficient of the mid-year population of women aged 15–49 of the survey year, based on United Nations population estimates [27] and divided by the survey sample size of women.

### Analysis

The study begins with descriptive analysis of TFRs and ASFRs in Egypt by single years from 1990 to 2013, examining how sub-groups of interest, especially women with secondary school or higher education and by place of residence, contributed to the increase in fertility rates. The user-generated Stata command tfr2 was used to compute ASFRs and TFRs by generating a table of exposure time and outcomes (live births) for single years from 1990 to 2013 by women’s five-year age groups [28]. Rates were adjusted by the de-normalized weight variable, and the standard errors, adjusted for clustering and stratification, of the ASFRs and TFRs were computed with jackknife by the tfr2 command. Individual exposure was multiplied by survey-specific all-woman inflation factors to adjust fertility rates for ever-married women and compute ASFRs and TFRs for all women of reproductive age.

While surveys were conducted every 2–3 years from 2000 to 2008, there was a six-year gap between the 2008 and 2014 surveys. Calculating fertility rates for women 15–49 during this period, using only the 2014 survey, resulted in the truncation of birth histories in the oldest age group (age 45–49). As fertility rates among Egyptian women age 45–49 tend to be low (2–7 births per 1000 women), and in order to generate comparable fertility rates for each year from 1990 to 2013, we calculated TFR for women aged 15–44 years.

Cross-sectional surveys such as the EDHS are limited in the calculation of real cohort fertility, or completed family size at age 50+. Instead, the mean number of children ever born by mother’s exact ages up to age 45 provides insight into patterns of childbearing and whether a change in the tempo or in the quantum, or both, is impacting the TFR. Mean parity at age 45 (due to EDHS sample of women under 50 years) can be interpreted as approximately equal to completed family size.

This study grouped all ever-married women into eight five-year birth cohorts to examine patterns of childbearing over time. By comparing age at first marriage and mean parity at exact ages, we sought to determine if a trend in more recent birth cohorts toward childbearing at younger ages might have contributed to the increased TFR recorded in the 2014 EDHS. Analysis focused on women who were at least 30 years or older at the time of interview as more than 90% of Egyptian women marry by age 30 [17]. Analysis of age at first marriage by women’s birth cohort while including young women (who necessarily had to have been married before the survey) results in a downward bias in estimates of age at first marriage. This is a particular issue among the most recent birth cohorts because they contain larger proportions of women who married at younger ages. For each five-year birth cohort from 1945–1949 to 1980–1984, the mean age at first marriage was calculated, stratified by place of residence and level of education at the time of the survey. To allow comparison of mean parity at exact ages to the values calculated for age at first marriage across the birth cohorts, analysis was limited to women age 30+. For mean parity at exact age 35, 40 and 45, only women age 35+, 40+ and 45+, respectively, at time of interview were included. Mean parity at exact ages included women who had not had a child by the age under consideration in the denominator.

### Data quality

Fertility estimates based on cross-section surveys of birth histories are subject to the respondent’s ability to accurately recall her own birthdate and that of her children, as well as the potential for bias in omission of birth information for children who have subsequently died [15]. All seven rounds of the EDHS considered here offer good quality data in terms of the completeness of month and year of birth information for women and their children, as any dates not provided by the respondent have been imputed [29]. While questions remain as to the accuracy of the birth dates provided and possible omission of births, successive rounds of the EDHS used in this paper allowed plotting of fertility trends in overlapping years to check for agreement [14,30].

### Ethical approval

The DHS receive government permission, use informed consent and assure respondents of confidentiality. The Research Ethics Committee of the London School of Hygiene and Tropical Medicine approved our analyses. Datasets used in this analysis are owned by The DHS Program, operated by ICF International.

## Results

### Results of data quality assessment

**Fig 1:**
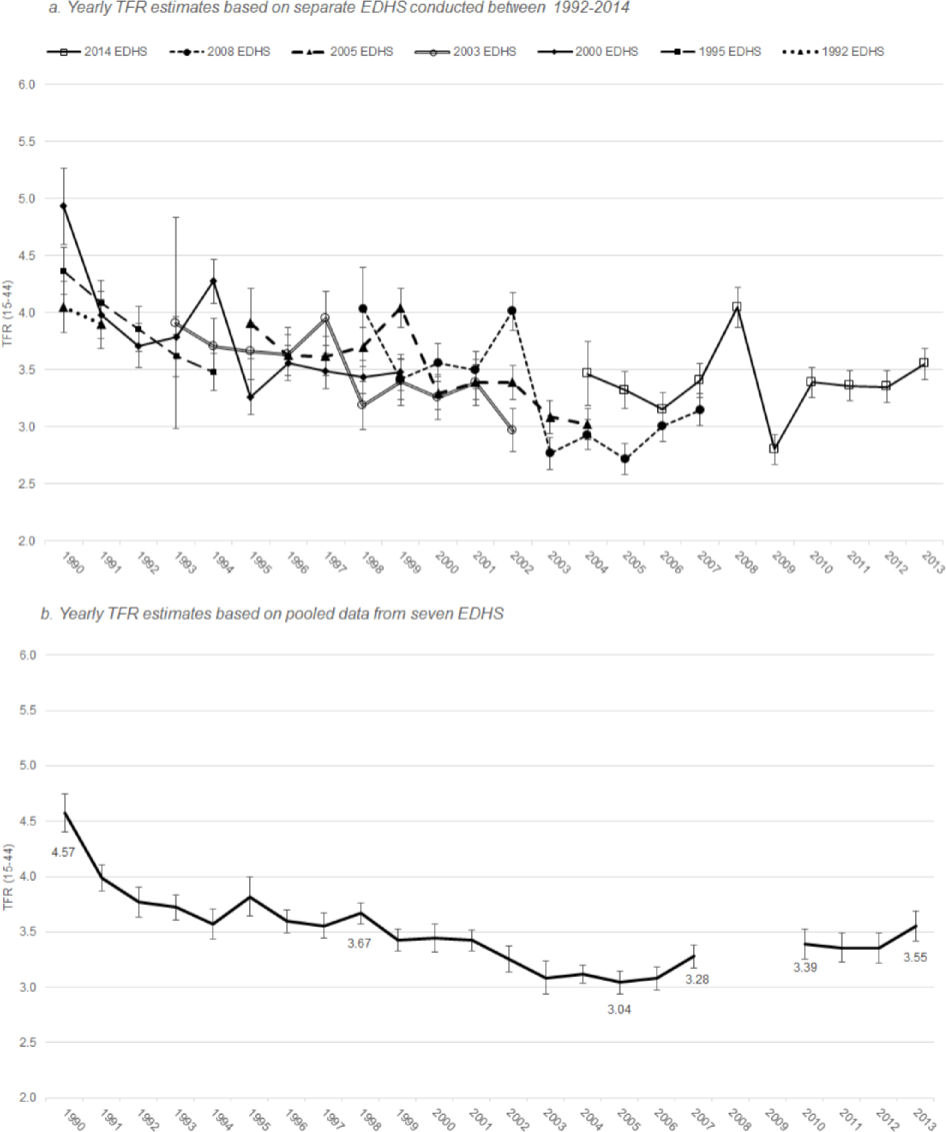
Annual TFRs (15–44), 1990–2013.

Birth history data up to 10 years before the interview for each of the seven surveys was used to calculate TFRs by calendar year from 1990–2013 to show the trends in fertility leading up to and immediately after the 2011 revolution. There is considerable variability in TFR estimates for single years when calculated from individual surveys, but as seen in Fig 1 (panel A), the confidence intervals overlap, except in the periods five and six years before each survey, where estimates are significantly lower or higher, respectively. Several studies have shown that enumerators sometimes omit or displace births to avoid completing the child health module for births within the last five years [30,31]. Due to this issue, the calculation of single-year fertility rates in the periods five and six years before each EDHS omit that survey. Thus, no TFR estimates were produced for the years 2008 and 2009 as these are five and six years before the 2014 survey. The fertility rate estimates for calendar years 2003 and 2010–2013 rely on only one survey (the 2005 EDHS and 2014 EDHS, respectively).

### Reconstructing fertility trends

Analysis of TFRs for women aged 15–44 using pooled data revealed that the steep fertility declines of the early 1990s were followed by slower overall declines until 2005. The general decline in fertility reversed in 2007, plateaued and then increased again in 2013 (Fig 1, panel B).

During the 24-year period under investigation, TFR reached its lowest point in 2005 at 3.04 (95% CI: 2.94–3.15). Compared to 2005, the TFR in 2007 increased significantly to 3.28 (95% CI: 3.18–3.38) and remained at levels comparable to 2007 until 2013, when it rose again to 3.55 (95% CI: 3.41–3.69). In 2013, the TFR was the highest since 1998.

**Fig 2:**
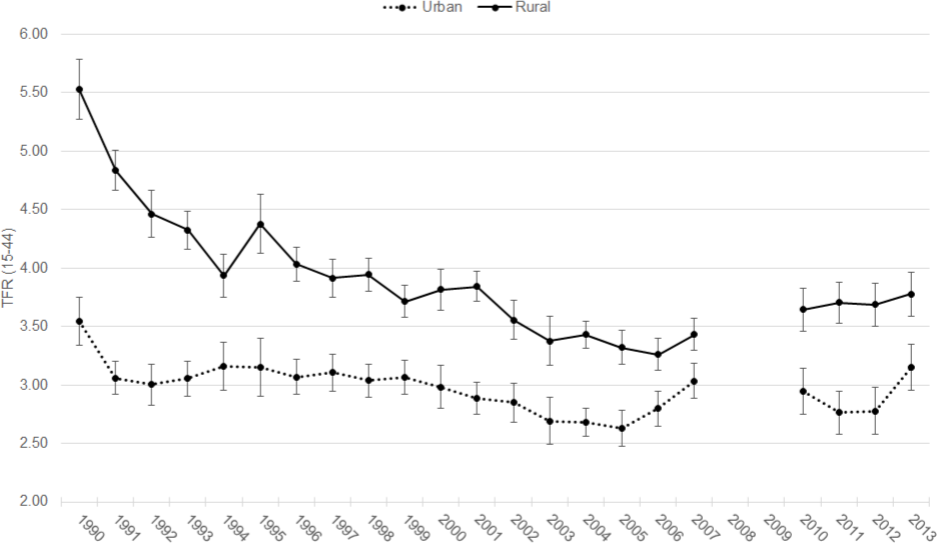
Annual TFRs by urban and rural residence, 1990–2013.

TFRs for urban women were lower than for rural women throughout the period under examination (Fig 2). Fertility among rural women declined dramatically from 5.03 (95% CI: 4.69–5.37) in 1990 to 3.94 (95% CI: 3.75–4.12) in 1994. This was followed by a much slower decline over the next 12 years to a low of 3.26 (95% CI: 3.12–3.40) in 2006, before reversing course. TFR for rural women then rose to 3.78 (95% CI: 3.59–3.96) in 2013—the highest level since 2001. Among urban women, fertility rates fluctuated but showed a slight overall decline to 2.63 (95% CI: 2.48–2.78) in 2005 before rising between 2006 and 2007 to 3.04 (95% CI: 2.88–3.19). From 2011 to 2012, urban fertility dropped below 3.0 before rising again in 2013 to 3.15 (95% CI: 2.95–3.35). A non-significant dip in fertility rates during and immediately following the 2011 revolution was observed in the Urban Governorates, which includes Cairo and Alexandria, but was less notable in urban Upper Egypt and not observed in urban Lower Egypt (results not shown).

**Fig 3:**
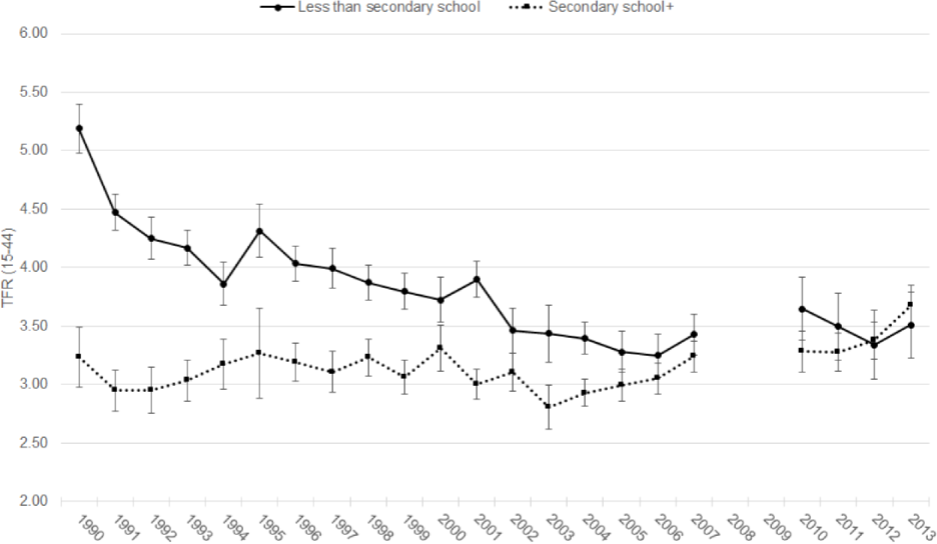
Annual TFRs by level of education, 1990–2013.

At the beginning of the period under examination, there was a clear divide in fertility levels between women with less than a secondary school education and those with secondary school or higher education (Fig 3). Secondary or higher educated women had nearly two children fewer than less educated women in 1990. Among less educated women, the TFR declined by 1.9 children in 16 years, from a high in 1990 of 5.19 (95% CI: 4.99–5.39) to a low in 2006 of 3.25 (95% CI: 3.09–3.40). Fertility then increased to 3.65 (95% CI: 3.44–3.86) in 2010 before declining slightly. Fertility among women with secondary or higher education showed little change from 1990–2006, fluctuating around a level of 3.0. From 2003 onward, fertility among women with secondary or higher education increased steadily, reaching 3.68 (95% CI: 3.49–3.87) in 2013, surpassing the TFR of less educated women. The increase in fertility among secondary school or higher educated women and the plateau in fertility rates among less educated women contributed to a convergence of fertility rates from 2005 onwards across the two levels of education.

**Fig 4:**
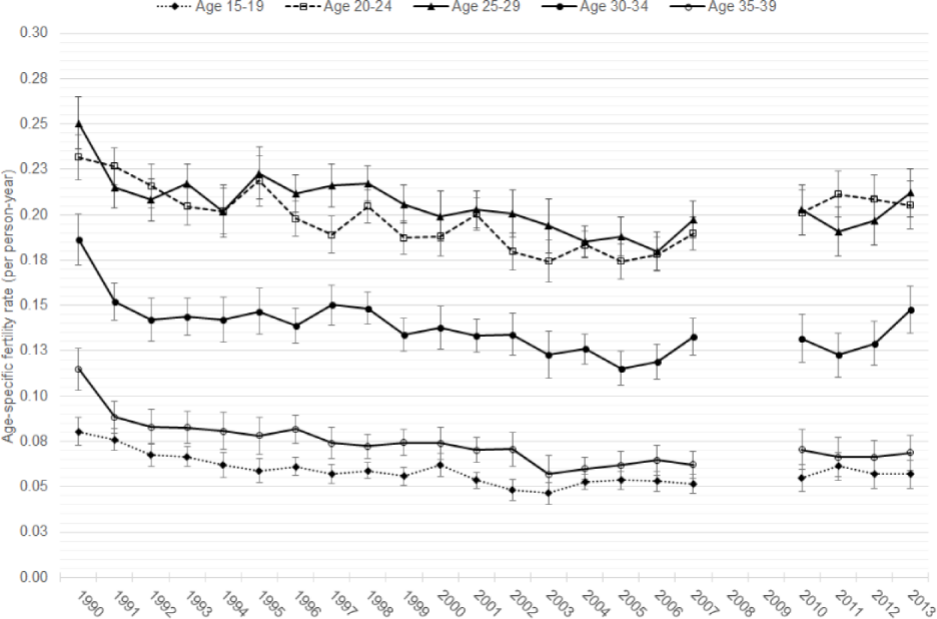
Annual ASFRs for women under age 40, 1990–2013.

Fig 4 shows age-specific fertility rates for women 15–39 by five-year age group. The secular rise in fertility after 2005 is seen clearly among 20–24 year olds, where rates increased from a low of 174 births per 1000 women (95% CI: 165–184) in 2005 to a high of 211 (95% CI: 199–224) in 2011. Fertility rates for women age 25–29 and 30–34 increased in 2007 from lows recorded in 2006 (for 25–29 year olds) and 2005 (for 30–34 year olds). ASFRs for these two age groups slightly declined in 2011 (though rates remained above pre-increase levels) compared to the 2007 increase, before rising again in 2013. Among women aged 30–34, ASFR increased to 148 per 1000 women (95% CI: 135–161) in 2013. The 2007 increase in TFR was observed among women age 20–34, and the 2013 increase was seen in sustained fertility rates above 2007 levels among 20–24 year olds and additional increases among women age 25–34. Fertility among 15–19 and 35–39 year olds declined until 2003 before plateauing. In 2011, fertility among 15–19 year olds briefly increased to 61 births per 1000 women (95% CI: 54–69) above the low of 46 births per 1000 (95% CI: 40–53) in 2003, before returning to 57 births per 1000 (95% CI: 49–65) in 2012–2013.

**Fig 5:**
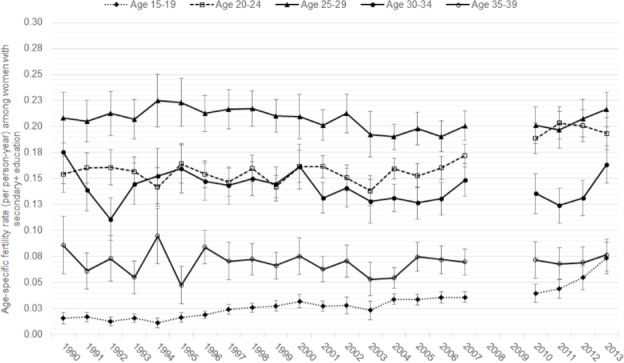
Annual ASFRs for women under age 40 with secondary or higher education, 1990–2013.

Fig 5 shows the annual ASFRs for women 15–39 by five-year age group among those with secondary or higher education. In contrast to the plateau observed among 15–19 year-olds in Fig 4, there was a clear increase in fertility rates in the second half of the study period among more educated women age 15–19. From 1990 to 2013, there was a nearly five-fold increase in fertility in this youngest, most educated age group. Among educated women age 20–24, fertility rates fluctuated around 150 births per 1000 until approximately 2005, where fertility began a slight but significant increase from 153 per 1000 (95% CI: 141–165) in 2006 to 193 per 1000 (94% CI: 178–209) in 2013.

### Patterns of childbearing by mother’s birth cohort

Ever-married women were grouped into five-year birth cohorts to explore changes in the patterns of childbearing through analysis of age at first marriage and mean children ever born by exact ages.

**Fig 6:**
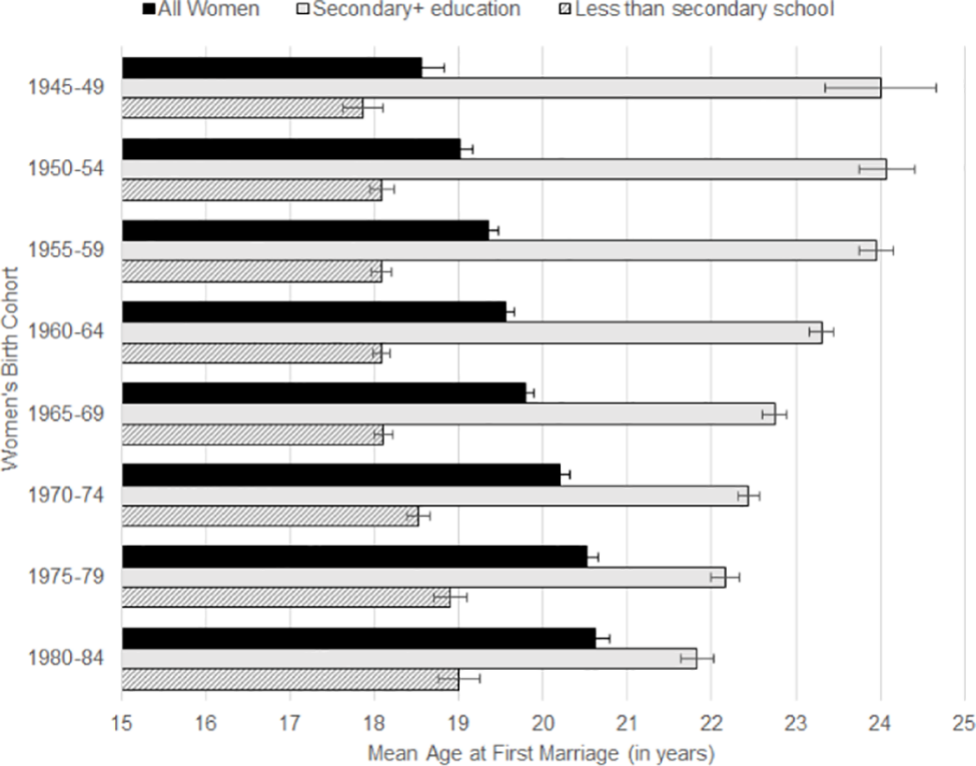
Mean age at first marriage by five-year birth cohort, comparing all ever-married women and stratified by level of education.

Mean age at first marriage was calculated by birth cohort for women age 30+ at the time of interview. Marriage age among all women increased by more than two years from 18.57 years (95% CI: 18.31–18.82) in the 1945–1949 cohort to 20.62 years (95% CI: 20.45–20.79) in the 1980–1984 cohort (Fig 6). Notably, the increase in marriage age from earlier to later birth cohorts is not seen among the subset of women with secondary or higher education. Quite the opposite, mean age at first marriage among these women peaked in the 1950–54 cohort at 24.08 years (95% CI: 23.75–24.41) and then decreased steadily in each subsequent birth cohort to 21.83 years (95% CI: 21.64–22.02) in the 1980–1984 cohort. Among women with less than a secondary school education, mean age at first marriage showed a slow increase of 1.2 years across the eight birth cohorts. The difference in mean age at first marriage among less educated women compared to those with secondary or higher education was more than six years in the oldest birth cohort (1945–1949) considered here. While more educated women married at older ages than less educated women across all the cohorts, this difference declined to less than three years in the 1980–1984 cohort.

Mean number of children ever born by women’s 20th, 25th, 30th, 35th, 40th and 45th birthdays was calculated for the 1945–1949 to 1980–1984 birth cohorts. Mean parity decreased at every exact age across the cohorts (Table 1). Mean parity at exact age 45 decreased by nearly two children from the 1945–1949 cohort to the 1965–1969 cohort, suggesting a true decline in completed family size across the five oldest birth cohorts. However, there is evidence that the pattern of childbearing shifted toward younger ages in the more recent birth cohorts considered here. In the 1945–1949 cohort, fertility before age 30 (measured as mean parity at exact age 30) accounted for 66% of completed family size (or mean parity at exact age 45). In comparison, the 1965–1969 cohort had lower mean parity at each exact age, but the proportion of fertility before age 30 accounted for 73% of this cohort’s completed family size. The larger contribution of fertility before age 30 for the 1965–1969 cohort led to a lower proportional increase in family size between exact ages 30 and 45 of 36%, compared to 52% in the 1945–1949 cohort. While the 1965–1969 cohort was having fewer children overall by age 45, a larger proportion of their childbearing occurred before age 30.

**Table 1:** Mean number of children ever born at mother's exact age by mother's five-year birth cohort.

	*Five-year birth cohort*							
	1945–49	1950–54	1955–59	1960–64	1965–69	1970–74	1975–79	1980–84
**Mean Parity by Age 20**^**a**^	0.95	0.76	0.68	0.64	0.60	0.49	0.44	0.38
(95% CI)	(0.88–1.01)	(0.73–0.79)	(0.65–0.70)	(0.62–0.66)	(0.58–0.62)	(0.47–0.51)	(0.42–0.46)	(0.35–0.41)
*Absolute increase*, *20 to 25*	*1*.*50*	*1*.*43*	*1*.*42*	*1*.*37*	*1*.*24*	*1*.*13*	*1*.*09*	*1*.*07*
*Proportional increase*, *20 to 25*	*158%*	*188%*	*209%*	*213%*	*204%*	*229%*	*248%*	*279%*
**Mean Parity by Age 25**^**a**^	2.44	2.19	2.10	2.01	1.84	1.62	1.53	1.45
(95% CI)	(2.34–2.54)	(2.13–2.25)	(2.05–2.14)	(1.97–2.05)	(1.81–1.87)	(1.59–1.65)	(1.49–1.57)	(1.40–1.50)
*Absolute increase*, *25 to 30*	*1*.*50*	*1*.*50*	*1*.*45*	*1*.*25*	*1*.*12*	*1*.*08*	*1*.*01*	*1*.*05*
*Proportional increase*, *25 to 30*	*61%*	*68%*	*69%*	*62%*	*61%*	*67%*	*66%*	*72%*
**Mean Parity by Age 30**^**a**^	3.94	3.69	3.55	3.26	2.96	2.70	2.54	2.51
(95% CI)	(3.82–4.07)	(3.62–3.76)	(3.49–3.60)	(3.22–3.31)	(2.92–3.00)	(2.66–2.73)	(2.50–2.58)	(2.45–2.56)
*Absolute increase*, *30 to 35*	*1*.*19*	*1*.*08*	*0*.*92*	*0*.*74*	*0*.*67*	*0*.*59*	*0*.*64*	
*Proportional increase*, *30 to 35*	*30%*	*29%*	*26%*	*23%*	*23%*	*22%*	*25%*	
**Mean Parity by Age 35**^**b**^	5.14	4.77	4.47	4.00	3.63	3.28	3.18	-
(95% CI)	(4.99–5.28)	(4.69–4.85)	(4.41–4.53)	(3.95–4.05)	(3.59–3.68)	(3.23–3.34)	(3.11–3.24)	
*Absolute increase*, *35 to 40*	*0*.*67*	*0*.*58*	*0*.*42*	*0*.*28*	*0*.*29*	*0*.*33*		
*Proportional increase*, *35 to 40*	*13%*	*12%*	*9%*	*7%*	*8%*	*10%*		
**Mean Parity by Age 40**^**c**^	5.80	5.35	4.89	4.28	3.92	3.61	-	-
(95% CI)	(5.64–5.97)	(5.25–5.44)	(4.81–4.96)	(4.21–4.34)	(3.85–3.99)	(3.53–3.69)		
*Absolute increase*, *40 to 45*	*0*.*21*	*0*.*27*	*0*.*07*	*0*.*15*	*0*.*11*			
*Proportional increase*, *40 to 45*	*4%*	*5%*	*1%*	*4%*	*3%*			
**Mean Parity by Age 45**^**d**^	6.01	5.62	4.95	4.43	4.04	-	-	-
(95% CI)	(5.83–6.19)	(5.49–5.75)	(4.85–5.06)	(4.32–4.54)	(3.93–4.14)			

^*a^Women aged 30+ at time of survey interview.

^b^Women aged 35+ at survey interview.

^c^Women aged 40+ at survey interview.

^d^Women 45+ at survey interview.

Among the most recent (1975–1979 and 1980–1984) birth cohorts, women reached approximately 2.5 children by exact age 30, despite the 1975–1979 cohort starting from a higher parity at exact age 20 compared to the 1980–1984 cohort. This suggests that the 1980–1984 cohort had more children between exact ages 20 and 30 in order to reach the same mean parity by age 30. For the 1980–1984 cohort, mean parity at ages 25 and 30 was lower compared to older cohorts, however the absolute difference in parity at these exact ages is slightly higher compared to the 1975–1979 cohort. This slight absolute increase represents a larger proportional increase between the two birth cohorts. Between ages 25 and 30, the 1980–1984 birth cohort saw a 72% increase in mean parity compared to the 66% increase during the same age interval in the 1975–1979 cohort. This is of interest because women born in 1980–1984 would have been in the age groups 20–24 and 25–29 in 2007—the first year documenting an increase in TFR—and these high fertility age groups both saw an increase in their respective ASFRs beginning in 2007.

Likewise, between exact ages 30 and 35, the 1975–1979 cohort saw slightly larger absolute increases in mean parity compared to the 1970–1974 cohort. Despite starting at a lower mean parity at age 30 of 2.54 (95% CI: 2.50–2.58) compared to the 1970–1974 cohort’s mean parity of 2.70 (95% CI: 2.66–2.73), women in the 1975–1979 cohort reached a similar mean parity at age 35 as the 1970–1974 cohort. Women in the 1975–1979 birth cohort would have been in their late 20s and early 30s during the 2007 fertility increase, and both 25–29 year olds and 30–34 year olds saw a rise in ASFRs starting in that year.

There is evidence of a shift in childbearing toward earlier ages among secondary or higher educated women in the more recent birth cohorts. In contrast to the declines in mean parity at age 45 observed in Table 1, among women with secondary or higher education, mean parity at exact age 45 remained the same across the five oldest birth cohorts (Table 2). However, the 1945–1949 birth cohort saw an 81% proportion increase in mean family size between exact ages 30 and 45, compared to a 56% proportional increase in the 1965–1969 cohort between the same ages. This suggests that the 1965–1969 cohort completed a larger proportion of childbearing before age 30, compared to the 1945–1949 cohort, in order for both cohorts to reach approximately the same mean parity at age 45.

**Table 2:** Mean number of children ever born to mothers with secondary or higher education by mother's five-year birth cohort.

	*Five-year birth cohort*							
	1945–49	1950–54	1955–59	1960–64	1965–69	1970–74	1975–79	1980–84
**Mean Parity by Age 20**^**a**^	0.09	0.07	0.05	0.06	0.10	0.11	0.15	0.19
(95% CI)	(0.05–0.14)	(0.04–0.09)	(0.04–0.07)	(0.05–0.07)	(0.09–0.12)	(0.10–0.12)	(0.13–0.17)	(0.16–0.21)
*Absolute increase*, *20 to 25*	0.50	0.51	0.61	0.79	0.88	0.90	0.94	0.97
*Proportional increase*, *20 to 25*	525%	785%	1155%	1259%	839%	830%	637%	516%
**Mean Parity by Age 25**^**a**^	0.59	0.58	0.67	0.85	0.98	1.01	1.09	1.15
(95% CI)	(0.46–0.73)	(0.51–0.64)	(0.62–0.71)	(0.81–0.89)	(0.95–1.02)	(0.97–1.04)	(1.04–1.13)	(1.10–1.21)
*Absolute increase*, *25 to 30*	*1*.*07*	*1*.*15*	*1*.*21*	*1*.*11*	*1*.*10*	*1*.*11*	*1*.*06*	*1*.*08*
*Proportional increase*, *25 to 30*	*180%*	*199%*	*182%*	*131%*	*112%*	*110%*	*98%*	*94%*
**Mean Parity by Age 30**^**a**^	1.66	1.73	1.88	1.96	2.08	2.11	2.15	2.24
(95% CI)	(1.49–1.83)	(1.63–1.82)	(1.82–1.94)	(1.92–2.01)	(2.04–2.13)	(2.07–2.16)	(2.09–2.20)	(2.18–2.29)
*Absolute increase*, *30 to 35*	*0*.*85*	*0*.*84*	*0*.*81*	*0*.*72*	*0*.*72*	*0*.*64*	*0*.*72*	*-*
*Proportional increase*, *30 to 35*	*51%*	*49%*	*43%*	*37%*	*35%*	*30%*	*34%*	
**Mean Parity by Age 35**^**b**^	2.51	2.57	2.69	2.68	2.81	2.75	2.87	-
(95% CI)	(2.34–2.68)	(2.47–2.67)	(2.62–2.76)	(2.63–2.74)	(2.75–2.86)	(2.69–2.82)	(2.79–2.94)	
*Absolute increase*, *35 to 40*	*0*.*43*	*0*.*39*	*0*.*34*	*0*.*37*	*0*.*36*	*0*.*35*	*-*	*-*
*Proportional increase*, *35 to 40*	*17%*	*15%*	*13%*	*14%*	*13%*	*13%*		
**Mean Parity by Age 40**^**c**^	2.94	2.96	3.03	3.05	3.16	3.11	-	-
(95% CI)	(2.74–3.13)	(2.84–3.07)	(2.95–3.12)	(2.99–3.12)	(3.08–3.24)	(3.01–3.21)		
*Absolute increase*, *40 to 45*	*0*.*07*	*0*.*03*	*0*.*05*	*0*.*10*	*0*.*10*	*-*	*-*	*-*
*Proportional increase*, *40 to 45*	*2%*	*1%*	*2%*	*3%*	*3%*			
**Mean Parity by Age 45**^**d**^	3.01	2.99	3.08	3.15	3.26	-	-	-
(95% CI)	(2.77–3.24)	(2.83–3.15)	(2.97–3.19)	(3.02–3.28)	(3.14–3.39)			

^*a^Women aged 30+ at time of survey interview.

^b^Women aged 35+ at survey interview.

^c^Women aged 40+ at survey interview.

^d^Women 45+ at survey interview.

Mean parity at age 30 for secondary or higher educated women was significantly higher in the 1965–1969 and later cohorts, compared to the 1945–1949 to 1960–1964 birth cohorts. Mean parity at age 25 has been steadily increasing from the 1955–1959 birth cohort onwards. From the 1955–1959 cohort to the 1980–1984 cohort, mean number of children by age 25 increased by 0.6 of a child among more educated women. This suggests that not only was fertility before age 30 increasing among women with secondary or higher education, but also that fertility in the early 20s and under age 20 has increased in the most recent cohorts. Mean parity at exact ages above 30 cannot be calculated for the 1980–1984 cohort as this cohort was age 30–34 at the time of the 2014 survey.

## Discussion

### Summary of findings

This study presented a comprehensive assessment of fertility in Egypt between 1990–2013 and is unique in using multiple rounds of EDHS to compute single-year fertility rates and its transparency in how the individual women’s observations captured by these surveys were weighted in the analysis. Combining multiple EDHS and using women’s birth histories to reconstruct fertility trends allowed further stratification by women’s age group and background characteristics due to the larger sample sizes.

Results suggest that fertility rates in Egypt first began to increase well before the 2011 revolution. After a low recorded in 2005 and beginning in 2007, there was a slow and steady increase in fertility rates seen in the highest fertility ages (20–34), in both urban and rural areas and among women with secondary or higher education. Due to poor data quality, fertility levels in 2008 and 2009 remain unclear. However, rates observed in 2007 and 2010 suggest a continuation or a slight increase in fertility between these years, followed by an additional increase seen in 2013. During the social upheaval and widespread protests in cities in 2011–2012, fertility declined among urban women but remained steady among rural women. These results suggest that the 2013 fertility increase was driven by continuing high fertility in rural women and a steeper rise observed in urban women from 2012 to 2013.

The notable convergence of TFRs in 2013 between women with and without secondary school education suggests that women’s education is no longer a strong predictor of fertility in Egypt. The increase in fertility rates and the decline in age at first marriage among secondary or higher educated women may be related to the changing socio-economic characteristics of the greater proportion of women with a secondary school education in the later surveys. While women’s education levels have increased in the more recent cohorts, their participation in the labor market has not kept pace [32,33]. The increase in fertility coincided with declines in public sector employment opportunities that are particularly appealing to women [32,34]. With few job prospects, educated women seem to have little incentive to delay marriage and childbearing.

From the older to more recent birth cohorts, age at first marriage increased slightly, shifting the onset of childbearing from late teens into early 20s. While there is evidence of declines in completed family size at age 45 in the younger birth cohorts, there is also evidence that the proportion of childbearing before age 30 is increasing in the more recent cohorts. This suggests a possible compression of childbearing between ages 20 and 30; however, it is too soon to determine whether these cohorts will continue in higher fertility regimes at older ages, leading to an increase in completed family size and thereby increasing fertility quantum.

Results suggest the pattern of childbearing among secondary or higher educated women shifted to slightly younger ages in more recent cohorts. Mean age at first marriage reached a peak for the secondary or higher educated women of the 1955–1959 cohort, and this coincided with a low in mean children ever born at exact ages 20 and 25. For the more educated women in birth cohorts after 1955–1959, there was a steady decline in mean age at first marriage and a steady increase in mean parity at age 20 and particularly at age 25. Childbearing before age 20 among educated women of the 1980–1984 cohort has returned to similar levels observed in the 1945–1949 cohort, though childbearing in the early and late twenties has increased. But the increase in childbearing during women’s early and late twenties may be plateauing; across the three most recent cohorts considered here, mean parity at age 30 among secondary or higher educated women appears to be stabilizing.

The increase in childbearing before age 30 among secondary or higher educated women in these most recent cohorts—at the same time as previous birth cohorts continued to have higher fertility rates at older ages—would operate to increase cross-sectional measures of fertility. This likely contributed to the slowing decline in TFR until 2005 and its increase thereafter. However, the uptick in ASFR among all women age 30–34 observed in 2013 suggests this may not be purely a concentration of childbearing (or an increase in tempo alone) before age 30. Time will tell if these more recent cohorts, who are having more children at younger ages, will continue in higher fertility regimes throughout their reproductive lives, leading to an increase in completed family size in this group.

### Limitations

The six-year gap between the 2008 and 2014 EDHS and the lack of good quality birth history information for 2008 and 2009 from the 2014 survey leaves the crucial years after the 2007 fertility increase unclear but does not significantly alter the main findings. While there has been an effort to adjust for enumerators’ omission and displacement of births in the five and six years before each survey in estimates of fertility rates, this remains a limitation in the analysis of parity at exact ages. The omission of some or all of the Frontier Governorates, which tend to have higher fertility rates, in three of the seven surveys may lead to a slight underestimation of fertility. Finally, this study is limited in its analysis of the extent to which tempo effects have contributed to a short-term uptick in TFR rather than an increase in completed family size due to the recent nature of the fertility change.

Finally, the EDHS interviewed only ever-married women, thus any births to unmarried women were not captured in the data. While likely few in number, the omission of these births may have led to a slight underestimate of fertility rates.

### Conclusion

In addition to changes in women’s employment opportunities, the increase in fertility and shift in the pattern of childbearing toward younger ages coincides with changes in the Egyptian government’s health policy priorities. Since the 1980s, USAID was Egypt’s major supplier of free or reduced cost contraceptive commodities—in particular the IUD [7,9] However, USAID had been scaling down support for family planning in Egypt since the 2000s, with free commodity distribution phased out by the end of 2007 [7,9,35]. The end of the USAID supported family planning project was part of a broader waning of political support for reproductive health in the final years of Mubarak’s regime in favor of a greater focus on curative care services. The Egyptian government continued to fund contraceptive distribution but support for mass media and incentives for family planning service providers declined.

This lack of political focus on family planning over the last decade may help explain the plateau in contraceptive use since 2003. After rapid increases in contraceptive uptake in the 1990s, contraceptive prevalence among married women age 15–49 has remained steady around 60% [7]. Between the 2008 and 2014 EDHS, use of IUDs declined while the proportion of women using the pill and injectable increased. While all are considered effective modern methods, there is evidence that women using the pill and other short-acting modern methods have shorter birth intervals, by choice or by contraceptive failure, compared to those using IUDs [36]. The shift in method mix between the two most recent EDHS may point to fundamental changes in family planning access—particularly for IUD insertion—following USAID’s phase out of subsidized contraceptive commodity distribution and provider training. Additional research is needed to examine changes in method mix and contraceptive use over time to try to understand how family planning access has changed over the last 10 years.

## Supporting information

S1 Fig Annual TFR (15–44) among women with no education, 1990–2013. Women with no education comprise a subset of women with less than secondary school education.
(EPS)
